# Estimating the Prevalence of Recreational Opioid Use in Spain Using a Multiplier Method

**DOI:** 10.3390/ijerph19084815

**Published:** 2022-04-15

**Authors:** José Pulido, Albert Sanchez-Niubo, Noelia Llorens, Juan Hoyos, Gregorio Barrio, Maria Jose Belza, Lucía Cea-Soriano, Ariadna Angulo-Brunet, Luis Sordo

**Affiliations:** 1 Departamento de Salud Pública y Materno-Infantil, Facultad de Medicina, Universidad Complutense de Madrid, 28040 Madrid, Spain; josepuli@ucm.es (J.P.); hoyosmiller@hotmail.com (J.H.); tlcea@ucm.es (L.C.-S.); lsordo@ucm.es (L.S.); 2 CIBER en Epidemiología y Salud Pública (CIBERESP), Instituto de Salud Carlos III, 28029 Madrid, Spain; gbarrio@isciii.es (G.B.); mbelza@isciii.es (M.J.B.); 3 Department of Social Psychology and Quantitative Psychology, Faculty of Psychology, University of Barcelona, 08035 Barcelona, Spain; 4 CIBER en Salud Mental (CIBERSAM), Instituto de Salud Carlos III, 28029 Madrid, Spain; 5 Delegación del Gobierno Para el Plan Nacional Sobre Drogas, Ministerio de Sanidad, Gobierno de España, 28008 Madrid, Spain; noelia.llorens@gmail.com; 6 Escuela Nacional de Salud, Instituto de Salud Carlos III, 28029 Madrid, Spain; 7 Faculty of Psychology and Education Sciences, Universitat Oberta de Catalunya, Rambla del Poblenou, 08018 Barcelona, Spain; ariadna.angulo.brunet@gmail.com

**Keywords:** opioid, overdose, prevalence, multiplier method, recreational opioid use

## Abstract

Acknowledgement of the prevalence of recreational opioid use (PROU) is key to the planning and evaluation of care services. However, in Spain, the prevalence of PROU in recent years is unknown. The objective of this study was to estimate the PROU between 2005 and 2019 in the general populations of six Spanish cities. A benchmark-multiplier methodology was used to estimate the PROU population size. The benchmark used was overdose deaths from recreational opioid use in Spain’s six most populated cities. The multiplier was the overdose death rate in a cohort of heroin users. Linear regression was used to estimate the trend of the PROU estimate over the set period of years. In 2005, the PROU was 4.78 (95%CI 3.16–7.91) per 1000 people. The estimated trend decreased, with the two lowest values being 2.35 per 1000 in 2015 and 2.29 in 2018. In 2019 the PROU was 2.60 per 1000 (95%CI 1.72–4.31), 45% lower than in 2005. While the decline in the PROU continues, its deceleration over the last four years calls for increased vigilance, especially in light of the opioid crisis in North America that has occurred over the last few years.

## 1. Introduction

Opioids are chemical substances that are used for both medical, primarily pain relief, and recreational purposes (in leisure contexts used without medical supervision) [1,2]. They are highly addictive, and their overdose (injury to the body that happens when an opioid is taken in excessive amounts) results in over 100,000 deaths each year around the world [1,2]. In recent decades, notable increases have been observed in the consumption of opioids in different parts of the world, especially in North America [1,3]. This increase, both in terms of therapeutic uses as well as recreational uses, has highlighted the need to improve surveillance systems in Spain and in other European countries [3], where opioid use (primarily heroin) was a leading public health problem in the 1980s and 1990s [4,5].

Surveillance of therapeutic use is carried out through reporting of the sales and medical prescription of opioids. In this sense, in Spain the use of medically prescribed opioids has increased in recent years [6], as was the case in the United States shortly before the opioid epidemic. Monitoring of recreational use of opioids is complex [7]. Since there are no registries of users, surveys are often used to determine the prevalence of consumption. One problem with population surveys is that they underestimate the prevalence of opioid use [8]. Similar to other illicit substances, users are less likely to accurately report their use. Nor do population surveys generally capture opioid use, since they are usually focused on the general public. Despite these limitations, surveys are used in Spain to monitor opioid consumption. These surveys suggest that the magnitude of the problem in recent decades is minor, with decreasing prevalence of use since 2000 [9,10]. We should not be limited to the results of surveys, however, whose results we know to be biased.

The inherent limitations in the use of surveys can be alleviated by using indirect methods (for example, the multiplier method, capture-recapture, etc.) [7,11]. These methods use indicators that can serve as a proxy to estimate the number of people who consume these sub-stances. The most commonly used indicators are those that focus on the consequences of use [8,12], such as records of detoxification treatment, hospitalizations and mortality [7,11]. With knowledge of these rates, we can use indirect statistical methods to infer consumption. These methods have been used in Spain in the past to estimate the prevalence of heroin use (the primary recreational opioid), though it should be noted that these estimates are outdated [8,12,13]. 

Therefore, given the limitations of using methods related to population surveys and the absence of more recent prevalence, this study aims to estimate the prevalence of recreational opiate use in Spain between 2005 and 2019. Specifically, we used opiate-related mortality indicator data from the six most populated Spanish cities and employed the multiplier method.

## 2. Materials and Methods

Mortality data in Spain were used to estimate the prevalence of recreational opioid users. Data on deaths due to recreational opioid use between 2005 and 2019 were collected from the drug-related mortality registry. This registry was created by the National Plan on Drugs in Spain [9], based on a general indicator created by the European Monitoring Centre for Drugs and Drug Addiction (EMCDDA) [14]. In this registry, the direct and fundamental cause of death is an acute, adverse reaction following non-medical and intentional use of psychoactive substances. The primary sources of information for this indicator are the Forensic Institutes, Forensic Doctors and the National Institute of Toxicology.

Deaths following recreational opioid use were selected with the following characteristics:

1. The existence of a history of opioid use immediately before death
2. Positive toxicological tests
3. Autopsy findings compatible with death due to acute reaction following opioid use
4. Forensic diagnosis of death due to opioid overdose

The following deaths were excluded:

1. Deaths without judicial intervention or forensic investigation
2. Deaths of persons under ten years of age or over 64 years of age
3. Deaths where the use of these substances may have been a contributing factor in the death but not the fundamental and direct cause of death
4. Deaths due to unintentional exposure or ingestion
5. Deaths due to adverse reactions to properly prescribed and administered psychoactive medications

Deaths that met the above criteria were included in the analyses. (Full inclusion/exclusion criteria in the Appendix A.1). In addition, following the recommendations of the EMCDDA, an analysis was performed that excluded those that met the criteria above and were also classified as suicide.

This registry has been collected since 2005 in the six most populous Spanish cities (Barcelona, Madrid, Seville, Valencia, Zaragoza and Bilbao; 2021 population: 7,522,583), which represent 16% of the total Spanish population, and 19% of those living in urban areas.

In addition to data on overdose deaths, we searched for an estimate of the overdose mortality rate (OMR). Only one study was identified for the data’s time and place of origin [15]. This study took place in Barcelona and Madrid with 791 heroin user participants aged 18–30 years who were followed up between 2001 and 2006. 

The multiplier method was used to estimate the PROU. This method requires an absolute figure as a benchmark and a related incidence rate (multiplier). From these two inputs, prevalence is estimated by multiplying the benchmark figure by the inverse of the multiplier [8].

In our case, the benchmark used was overdose deaths (OD) from recreational opioid use. The multiplier was the OMR in a cohort of heroin users. So if OMR = OD/PROU, PROU = OD/OMR was calculated. 

Confidence intervals for prevalence were estimated a posteriori by calculating percentiles from a distribution constructed using the bootstrap method [16]. This distribution was based on a normal distribution, with the mean as the point estimate of the mortality rate and the standard deviation as an approximation using the variability of the rate. Thus, one million prevalence estimates were calculated using random values under the normal distribution of the mortality rate. These estimates were then ordered from lowest to highest, and the 2.5% and 97.5% percentiles were identified as the confidence intervals of the prevalence estimate.

Once the PROU and its variability were estimated for each year, the linear trend was calculated over the set period of years using a simple linear regression and standardized by the total population of each year.

## 3. Results

In 2005, the number of deaths due to recreational opioid use in the 6 Spanish cities was 182 (15 of them due to suicide), implying a mortality rate of 0.025 deaths per 1000 inhabitants. From this figure, the prevalence of recreational opioid use was estimated to be 4.78 per 1000 inhabitants (95% CI 3.16–7.91) without taking suicides into account. 

Until 2014 there was a decline in the prevalence estimate, which that year was 2.45 per 1000 (95% CI 1.62–4.06). From then to 2019, the figures had remained more or less stable between 2.29 in 2018 and 2.64 in 2016. The overall number of suicides ranged from 7 to 22. However, there were no significant differences in the PROU estimates of whether suicides were taken into account (Table 1).

**Figure 1:**
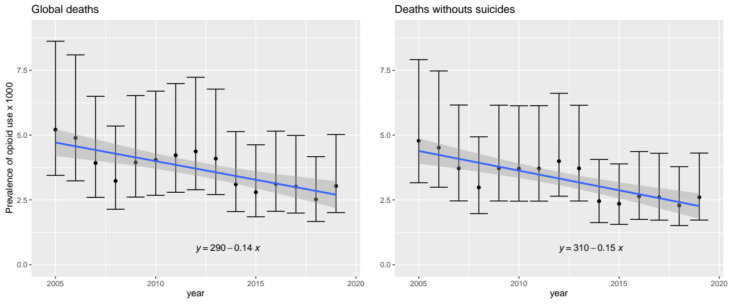
Estimation of recreative opioid use rate evolution in the six most populated cities of Spain (2005–2019).

Overall, from 2005 to 2019, the estimated PROU trend is slightly decreasing. The PROU estimate in 2019 is 2.60 per 1000 (95% CI 1.72–4.31), 45% lower than in 2005 (Figure 1). 

## 4. Discussion

This study estimated the prevalence of recreational opioid use in six of Spain’s most populous cities using the multiplier method based on death records. The PROU estimate decreased from 4.78 per 1000 inhabitants in 2005 to 2.60 in 2019. However, this decrease showed some slowing in the last four years.

The comparison and contextualization of the results of this study is a complex task. On the one hand, the figures derived from the Spanish National Drug Plan surveys (carried out every two years) [9] show that since 2005, 0.1% of Spanish people aged 15 to 64 have taken heroin in the last 12 months. These figures are lower than those estimated in this study. However, they are questionable because they are based on a fixed address population. Our findings are consistent with those reported in other studies carried out in Spain. Prevalence of consumption between 0.5% and 0.8% were reported in 2005 [4,5], which are somewhat higher than ours but were calculated for people under age 44. In addition, a decline in use was reported during the 2000–2009 time period [13], which is similar to that reported in this study. Looking at opioid treatment indicators, there was an apparent decrease from the 1990s until 2008 and a slighter decrease since 2009 [9]. 

In addition, other indirect data showed reductions along the same lines as our data, such as the decrease in urgent hospital admissions for recreational opioid use between 2013 and 2016 [17] and the detection of heroin in wastewater between 2011 and 2015 [18]. All of this has taken place in an urban context, in which the norm is greater use of substances. Thus, these figures should only be extrapolated to the whole of Spain with much caution.

One of the points most in need of discussion in this article is the assumptions made regarding the method in which performance is uncertain unless strong ‘population steady state’ assumptions are made [19,20]. The benchmark, deaths due to recreational use of opioids, is exhaustive. The city-based registry was chosen for its consistency and reliability over the years [9]. The rate used represents the population, given that it is a cohort of the same time period and was drawn from two of the cities. This rate represents people who used opioids not necessarily in treatment, and it was obtained independently of the benchmark [15]. The fact that the people are heroin users represents the reality in Spain. We cannot know how much of what we call recreational opiates is accounted for by heroin. However, all of the studies conducted in Spain indicate that the recreational use of opioids has referred almost entirely to heroin users during both the years of the study and in recent years [6,21,22]. 

One problem is that the rate calculated between 2001 and 2006 was applied for the entire study period. However, there is no other current rate that can be used, and there is no reason to think that the rate may have changed in recent years in Spain: there is no evidence of changes in odds of overdoses [23] due to, for instance, synthetic opioids such as fentanyl, as it has been described in other countries [24] or patients shifting to black-market opioids when they no longer could be supplied with legal product opioids. This is why this method can be applied in this context and at this time. In any case, this paper recommends making updates to the mortality rates of this population.

## 5. Conclusions

The results of this study provide estimates that have not been available for the last decade in Spain. Up to 2019, it does not seem that there has been a scenario comparable to that of North America in terms of the recreational opioid crisis [6]. However, both the international context and the figures from recent years suggest the need for extreme vigilance. The use of indirect methods is excellent for estimating hidden populations.

## Figures and Tables

**Table 1:** Estimation of the prevalence of recreative opioid use in the six most populated Spanish cities.

	Number of Deaths					Estimation of PROU/1000			Estimation of PROU/1000 without Suicides		
Year	Number in Register	Suicides	Ref. Population	% Spanish Population	Deaths/1000	Point	95% LCL	95% UCL	Point	95% LCL	95% UCL
2005	182	15	7,251,688	16.75	0.0251	5.21	3.45	8.62	4.78	3.16	7.91
2006	170	13	7,249,252	16.47	0.0235	4.89	3.24	8.10	4.52	2.99	7.48
2007	136	7	7,233,937	16.15	0.0188	3.92	2.60	6.50	3.72	2.46	6.16
2008	103	8	6,657,856	14.58	0.0155	3.23	2.14	5.35	2.98	1.97	4.93
2009	140	8	7,426,081	16.06	0.0189	3.94	2.61	6.53	3.72	2.46	6.15
2010	143	12	7,436,169	16.00	0.0192	4.04	2.68	6.69	3.71	2.45	6.13
2011	148	18	7,410,976	15.88	0.0200	4.22	2.79	6.99	3.71	2.45	6.14
2012	152	13	7,387,118	15.78	0.0206	4.37	2.89	7.23	4.00	2.64	6.61
2013	141	13	7,344,914	15.72	0.0192	4.09	2.71	6.78	3.72	2.46	6.15
2014	105	22	7,265,367	15.62	0.0145	3.10	2.05	5.13	2.45	1.62	4.06
2015	94	15	7,238,722	15.58	0.0130	2.80	1.85	4.63	2.35	1.55	3.89
2016	105	16	7,263,300	15.64	0.0145	3.11	2.06	5.15	2.64	1.75	4.37
2017	102	14	7,293,097	15.67	0.0140	3.01	1.99	4.98	2.60	1.72	4.30
2018	86	8	7,338,520	15.73	0.0117	2.52	1.67	4.17	2.29	1.51	3.78
2019	105	15	7,407,608	15.75	0.0142	3.04	2.01	5.02	2.60	1.72	4.31

## Data Availability

Reference populations were obtained from the Spanish National Institute of Statistics.

## Appendix A. Full Inclusion and Exclusion Criteria for Recreational Opioid Overdose Deaths

### Appendix A.1. Stage One

Examine whether or not the case meets any of the EXCLUSION CRITERIA. If yes, it is discarded and not recorded. If not, the case is moved to the second stage. The exclusion criteria are described as follows:

(a) Deaths without judicial intervention or forensic study on their causes with a written record of the results.
(b) Deaths of people under age 10 or over age 64 years.
(c) Deaths NOT related to the use of psychoactive substances (cancer, diabetes mellitus, pneumonia, etc.) However, deaths due to pathologies that may have been aggravated or complicated by the recent use of psychoactive substances, such as sudden and unexpected deaths due to cardiovascular or neurological disease, ARE NOT EXCLUDED and move to stage two for further consideration.
(d) Deaths indirectly related to the use of psychoactive substances, that is, those in which the use of these substances may have been a contributing factor to death, but not the fundamental and direct cause of death, such as deaths due to:
  - Infectious pathologies (AIDS, endocarditis, tuberculosis, hepatitis, etc.), even if related to the use of psychoactive substances.
  - Homicide.
  - Accidents of any type (occupational, domestic, traffic, etc.) in persons under the influence of psychoactive substances are excluded; however, deaths caused directly by poisoning, or acute intoxication with these substances are moved to stage two.
  - Suicide of any type (hanging, precipitation, immersion, injury by firearm, etc.) in persons under the influence of psychoactive substances, EXCEPT deaths caused directly by poisoning or acute intoxication with these substances.
(e) Deaths (for which there is obvious evidence) that are due to involuntary or unintentional exposure to or ingestion of psychoactive substances. For example, deaths from a broken bag of drugs ingested for the purpose of trafficking or occupational exposure to a recordable volatile substance. Consumption resulting from the existence of dependence would be considered voluntary or intentional.
(f) Deaths due to adverse reactions to properly prescribed and administered psychoactive drugs.
(g) Deaths due to chronic diseases related to alcohol consumption and deaths due to acute alcohol intoxication exclusively (binge drinking), without evidence of acute reaction to other recordable psychoactive substances.
(h) Deaths due to the use of volatile substances, household products or caustics, EXCEPT when inhaled or sniffed.

### Appendix A.2. Second Two

Cases that have not been discarded are examined in terms of the INCLUSION CRITERIA. If a case does not meet any of them, it will be discarded and not registered. If a case meets at least one, it will be registered. The inclusion criteria are described as follows:

(a) History of recent psychoactive substance use.
  - Documented clinical evidence (hospital records or reports, etc.), of acute pathology due to psychoactive substance use immediately prior to death.
  - Physical signs of recent administration of psychoactive substances or presence of traces of psychoactive substances in the mouth, nostrils, stomach, etc.
  - Presence of psychoactive substances or utensils (syringes, foil, pill bottles, etc.) at the place of death.
  - History of recent consumption described by relatives or friends or collected by a coroner with forensic medical-legal expertise shortly before death.
(b) Positive toxicological tests for any of the psychoactive substances that can be registered.
(c) Autopsy signs compatible with death by acute reaction after consuming psychoactive substances.
(d) Forensic diagnosis of death by the acute reaction after consumption of psychoactive substances (overdose, etc.).
